# Survival and recurrence with or without axillary dissection in patients with invasive breast cancer and sentinel node metastasis

**DOI:** 10.1038/s41598-021-99359-w

**Published:** 2021-10-06

**Authors:** Vanessa Monteiro Sanvido, Simone Elias, Gil Facina, Silvio Eduardo Bromberg, Afonso Celso Pinto Nazário

**Affiliations:** 1 Breast Surgery Team, Department of Gynecology, Universidade Federal de São Paulo (UNIFESP), Rua Napoleão de Barros 608, Vila Clementino, São Paulo, SP CEP 04024-002 Brazil; 2 Hospital do Coração (HCor), São Paulo, Brazil; 3 Hospital Israelita Albert Einstein (HIAE), São Paulo, Brazil

**Keywords:** Breast cancer, Surgical oncology

## Abstract

To evaluate overall survival and locoregional recurrence between patients with invasive breast tumours and sentinel node metastasis undergoing sentinel lymph node dissection (SLND) alone and those undergoing complete axillary lymph node dissection (ALND). In this retrospective cohort study, we reviewed the medical records of patients with invasive breast carcinoma who underwent lumpectomy at a public university hospital in Brazil between 2008 and 2018. We evaluated the overall survival and the locoregional recurrence using Kaplan–Meier and Cox regression analyses, respectively. Overall, 97 participants who underwent lumpectomy were enroled; 41 in the ALND group, and 56 in the SLND group, according to Z0011 criteria. Only 17% of the patients in the ALND group had an additional biopsy-proven axillary disease, and 83% were treated with complete dissection unnecessarily. The 5-year survival rates were 80.1% and 87.5% for SLND and ALND, respectively (p = 0.376). Locoregional recurrence was rare (1.7% and 7.3% in the SLND and ALND, respectively; p = 0.3075). Overall survival and locoregional recurrence were similar between the two groups. The de-escalation of ALND to SLND in women with metastasis in the sentinel lymph node treated with conservative surgery and radiotherapy that meet the Z0011 criteria is feasible even in developing countries.

## Introduction

Breast cancer is the most common type of cancer worldwide and is responsible for 15% of cancer-related deaths among women^1^. Sociodemographic index levels are significant determinants of breast cancer incidence and mortality. In low- and middle-income countries, the incidence rate of breast cancer is low^2^. However, breast cancer mortality remains high due to limitations in early diagnosis and treatment options^3,4^.

In recent decades, the standard axillary management of early-stage breast cancer has changed dramatically. Axillary lymph node dissection (ALND) has been replaced by sentinel lymph node dissection (SLND) for the treatment of clinically negative lymph node breast cancer^5^. The American College of Surgeons Oncology Group (ACOSOG) Z0011 was a milestone in the surgical treatment of the axilla in patients with early breast cancer, and it significantly contributed to reducing the extent of breast surgery^6–11^. Consequently, the omission of ALND resulted in reduced morbidity and improved quality of life^12–15^.

ACOSOG Z0011 was a randomized, and non-inferiority clinical trial on women with invasive breast tumours measuring up to 5 cm with clinically negative axilla and up to two metastatic sentinel lymph nodes (SLNs). Patients were treated with lumpectomy, breast radiotherapy, and systemic adjuvant therapy. This study showed that completion of ALND neither significantly improved overall survival or disease-free survival nor did it reduce locoregional recurrence in these patients^15,16^. The ACOSOG Z0011 data were updated with a 10-year follow-up, and the results confirmed the evidence, leading to fundamental changes in the surgical management of the axilla^17,18^.

Other studies have corroborated the results of the Z0011trial. NSABP B4 found no benefit with resection of positive occult lymph nodes at the time of surgery^19^. IBCSG 23-01 also evaluated patients with minimal lymph node involvement and showed that ALND could be avoided^20^. The AMAROS trial confirmed that axilla treatment (surgery or radiotherapy) in patients with metastatic axillary SLNs provides comparable axillary control with no significant differences in overall survival between treatment groups. Additionally, axillary radiotherapy can be used as an alternative to ALND in patients with metastasis in the SLN, which fulfils the ACOSOG Z0011 exclusion criteria^21^.

However, several shortcomings were identified in the Z0011 trial, such as unmet accrual goal in enrollment, absence of standard testing for human epidermal growth factor receptor 2 (HER2), doubts about the radiotherapy fields, and applicability to other patient populations^22^.

In Brazil, the conservative approach to the axilla in patients with positive SLN biopsies has been challenged because survival after breast cancer remains poor, as in other developing countries; this is probably due to diagnosis of the disease at more advanced stages^23,24^. In addition, there is resistance in some cancer centres to adhere to the new surgical approach. These facts motivated this study.

This study aimed to validate the applicability of the Z0011 trial approach by evaluating the overall survival and locoregional recurrence in patients with invasive breast cancer who underwent either SLND as indicated by biopsy or complete ALND.

## Materials and methods

### Study design, setting, and ethics

This was a retrospective cohort study based on medical records evaluating the survival in consecutive patients with primary invasive carcinoma of the breast who underwent conservative surgery at a public university hospital in Brazil (Hospital São Paulo, Universidade Federal de São Paulo, UNIFESP) between February 2008 and December 2018. We evaluated overall survival and locoregional recurrence in patients who underwent either SLND or ALND.

This project was approved by the institutional review board (Comitê de Ética em Pesquisa da Universidade Federal de São Paulo—UNIFESP) under number (1.727.717/2016). Informed consent was waived for this observational study, patients signed informed consents for each procedure individually and the study did not pose any additional risk or discomfort for patients. Anonymity was guaranteed. This study is reported according to the STROBE Statement (STrengthening the Reporting of Observational studies in Epidemiology) reporting guideline and is in accordance with the Declaration of Helsinki.

### Participants, sources of data, and treatments received

We reviewed an electronic database of the surgical ward records to identify patients (only women) who underwent surgery during the study period for a primary breast tumour measuring up to 5 cm. We excluded patients who underwent a mastectomy, complete axillary lymph node resection without a previous SLN biopsy, and those who received neoadjuvant therapy. We considered only women who underwent conservative breast surgery and SLN biopsy. Next, we excluded patients who had negative sentinel lymph node biopsy test findings. Therefore, only those who underwent conservative surgery (lumpectomy) and had positive sentinel lymph nodes were finally included. The axillae of these women were clinically negative (N0).

**Figure 1:**
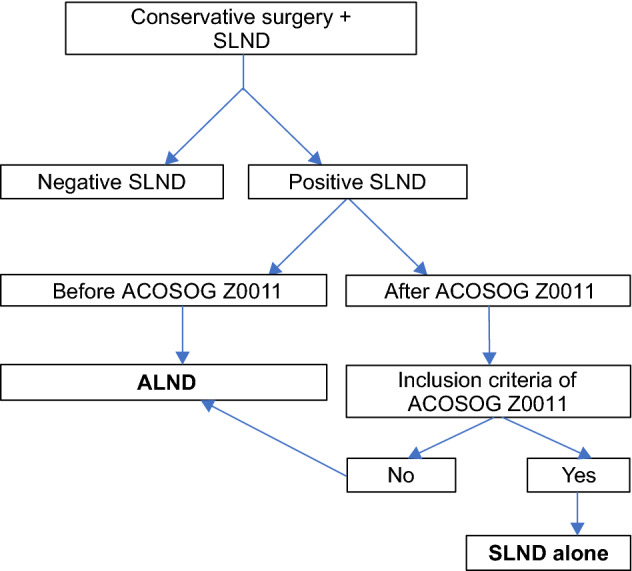
Clinical approach for the treatment of the axilla after the publication of the Z0011 trial. *SLND* sentinel lymph node dissection, *ALND* complete axillary lymph node dissection.

We divided the participants into two groups: the ALND group, which included patients who underwent complete ALND, and the SLND group, which included those who underwent dissection of only the axillary nodes indicated by the SLN biopsy. Figure 1 shows the treatment protocols for patients at our hospital before and after the publication of the Z0011 trial. All patients in the SLND group met the Z0011 criteria and ALND can be omitted. The Z0011 criteria included women with clinical T1-T2 invasive breast cancer, no palpable adenopathy, and 1 to 2 SLNs containing metastases, who underwent lumpectomy and tangential whole-breast irradiation^15^.

The only Z0011 trial criterion that was not considered at our hospital was the presence of extracapsular extension identified as focal or smaller than 2 mm. These patients were treated with SLND. Patients with Z0011 exclusion criteria were treated with ALND.

During the study period, ultrasonography was not routinely performed at our hospital. Sentinel lymph node biopsy was performed using patent blue dye, while some patients were treated using a combined technique with technetium-99m. Lymphoscintigraphy was not routinely performed because of the lack of resources at our hospital. Intraoperative assessment of SLNs could be performed during surgery at the surgeon's discretion.

### Study endpoint, variables, and sample size calculation

We compared survival between the groups. The primary endpoint was overall survival, which was defined as the time between surgery and death for any cause. Secondary endpoints were locoregional recurrence, which was determined by the return of the disease either in the breast, ipsilateral lymph nodes in the axilla, or in the internal supraclavicular, infraclavicular, or thoracic (mammary) chains and survival after the recurrence of the disease, which was considered as the time between surgery and the onset of locoregional recurrence.

We also compared sociodemographic and clinical variables such as age group (younger than 50 years or aged 51 years or older), race, educational level, histological diagnosis and grade, angiolymphatic invasion, hormonal receptors, HER-2 positivity, Ki-67, tumor size, number of positive lymph nodes, and therapy between the groups.

The sample size was calculated to detect the non-inferiority of risks (hazard ratio) with a power of 85% and a significance level of 5%. We admitted a non-inferiority margin of 0.30 for the risks and a follow-up of 5 years, in which we assumed an approximately exponential distribution. A minimum sample size of 43 patients for each group (total, 86 patients) was determined. We used PASS 14 software (Power Analysis and Sample Size System, NCSS) for this calculation.

### Statistical analysis

We analyzed the data descriptively. For categorical variables, we present absolute and relative frequencies and, for numerical variables, summary measures (mean, quartiles, minimum, maximum, and standard deviation).

We verified associations between two categorical variables using a Chi-square test or in cases of small samples with a Fisher's exact test. We compared two means using a student’s t-test for independent samples, with the assumption of normality verified using a Kolmogorov–Smirnov test.

The survival analyses in this study evaluated the time until the occurrence of death or recurrence, considering censorship (cases that did not experience the event during the analysis period). Initially, we analyzed survival functions separately for each predictor variable (univariate analysis). Kaplan–Meier models were used for categorical variables. We estimated survival functions for each level of these variables, and then compared them using a log-rank test (Mantel–Cox).

Additionally, we adjusted univariate Cox regression models for all the predictor variables considered, and a multivariate Cox model was adjusted for significant characteristics identified in the univariate models (backward method). A Cox's model assumes the existence of proportional risks, which was verified via a test based on Schoenfeld residuals.

Locoregional recurrence rates were compared with the Fisher’s exact test. All statistical tests were two-tailed, and a significance level of 5% was used. Statistical analyses were performed using the statistical software Statistical Package for the Social Sciences (SPSS) 20.0 and STATA 12.

### Ethics approval and consent to participate

The Institutional Review Board of Universidade Federal de São Paulo, number 1.727.717/2016, approved this project. All the procedures of the study were in accordance with the ethical standards of the Helsinki declaration. Patients were informed about the study objectives and agreed to have their clinical data used in the research without any incentive, signing an informed consent form. 

## Results

### Patient characteristics

**Figure 2:**
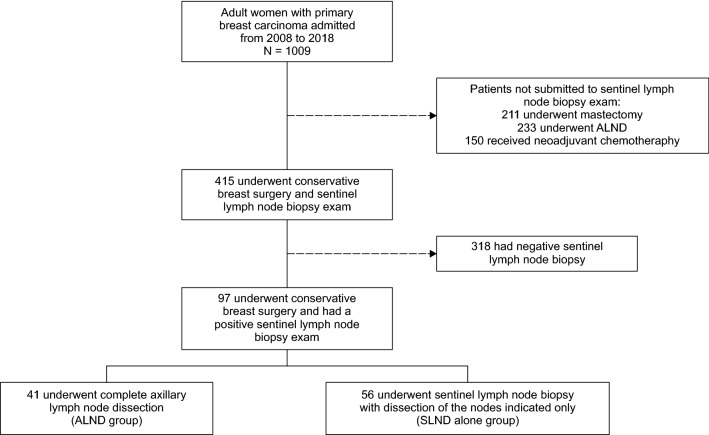
Flow diagram of patient inclusion procedure used in the study.

During the study period, 1009 patients underwent surgery for breast cancer at our hospital. After applying the exclusion criteria, we identified 97 patients with positive lymph nodes who underwent conservative breast surgery. These patients were diagnosed with invasive breast carcinoma, with tumours of 2 cm or less (T1) or 2.1 cm to 5 cm (T2), all with clinically negative axilla (N0). There were 41 and 56 patients in the ALND and SLND groups, respectively (Fig. 2).

The mean age was similar between the groups (Table 1). The mean tumour size was 1.7 cm, and it was significantly larger in the ALND group compared with that in the SLND group (p = 0.048). The number of positive lymph nodes was significantly higher in the ALND group (median, 2) than in the SLND group (median, 1). Only one lymph node was positive in 91% of the cases. In the SLND group, all patients had one or two positive lymph nodes. In the ALND group, only 17% of patients had an additional axillary disease (as shown by biopsy), suggesting that the complete dissection performed in 83% of patients was unnecessary. The ALND group had a significantly higher number of total lymph nodes removed (median, 14) than the SLND group did (median, 2). The ALND group also had a significantly longer follow-up time than the SLND group did (median, 5.3 vs. 3.5 years).

**Table 1:** Baseline clinical characteristics and follow-up in patients with breast cancer.

	Mean	Standard deviation	Minimum	Maximum	First quartile	Median	Third quartile	N	p*
**Age**	**57.8**	**11.5**	**29.0**	**93.0**	**49.0**	**58.0**	**66.0**	**415**	0.456
SLND alone	58.3	12.3	34.0	84.0	49.3	58.0	67.8	56	
ALND	56.3	12.8	33.0	77.0	46.0	58.0	66.5	41	
**Tumor size (cm)**	**1.7**	**1.1**	**0.0**	**6.2**	**1.0**	**1.5**	**2.3**	**415**	0.048
SLND alone	1.8^B'^	0.9	0.0	4.5	1.2	1.7	2.3	56	
ALND	2.2^A'^	1.1	0.1	4.5	1.4	2.0	3.1	41	
**Positive lymph nodes**	**0.5**	**1.8**	**0.0**	**24.0**	**0.0**	**0.0**	**0.0**	**415**	0.002
SLND alone	1.1^B'^	0.3	1.0	2.0	1.0	1.0	1.0	56	
ALND	3.5^A'^	4.5	1.0	24.0	1.0	2.0	4.0	41	
**Resected lymph nodes**	**3.9**	**4.5**	**1.0**	**30.0**	**1.0**	**2.0**	**4.0**	**415**	< 0.001
SLND alone	2.7^B'^	2.1	1.0	12.0	1.0	2.0	4.0	56	
ALND	14.5^A'^	5.4	5.0	30.0	11.5	14.0	18.0	41	
**Immunohistochemistry (ki67)**	**23.3%**	**20.3%**	**1.0%**	**90.0%**	**10.0%**	**20.0%**	**30.0%**	**379**	0.519
SLND alone	22.8%	20.3%	5.0%	80.0%	10.0%	14.5%	30.0%	54	
ALND	25.6%	17.9%	5.0%	90.0%	10.0%	20.0%	38.8%	32	
**Follow-up (years)**	**4.3**	**2.5**	**0.1**	**13.8**	**2.3**	**4.2**	**5.8**	**415**	0.012
SLND alone	3.7^B^	1.9	0.8	8.1	2.0	3.5	5.0	56	
ALND	5.0^A^	2.9	0.8	10.4	2.2	5.3	7.4	41	

Bold values indicates category headings and the total value of the category by group.

(A) and (B) are different means according to the multiple comparisons of Duncan.

(A') and (B') are different means according to the multiple comparisons of Dunn–Bonferroni.

*ALND* complete axillary lymph node dissection, *SLND* sentinel lymph node dissection.

*Student's t test for comparison of mean values between two groups.

There was no significant difference in race, educational level, histological diagnosis and grade, hormonal receptors, HER-2 positivity, tumour size (according to TNM pathological staging), and angiolymphatic invasion between the groups (Table 2). However, the ALND group had a significantly higher number of positive lymph nodes and macrometastases (p < 0.001 for both), as well as higher axillary involvement, as seen by the higher frequency of pN1, pN2, and pN3 cases (p < 0.001). In contrast, the SLND group had less extracapsular extension (p < 0.001). Adjuvant chemotherapy was more frequent in the ALND group (p = 0.024). Furthermore, the types of radiotherapy and hormonal therapy performed were similar between the groups.

**Table 2:** Demographic and clinical characteristics of the patients.

	Group				**p**
	SLND		ALND		
	N	%	N	%	
**Race**	**56**	**100.0%**	**41**	**100.0%**	**0.120**^**a**^
White	47	83.9%	28	68.3%	
Mixed	7	12.5%	10	24.4%	
Black	1	1.8%	3	7.3%	
Other	1	1.8%	0	0.0%	
**Educational level**	**49**	**100.0%**	**41**	**100.0%**	**0.142**^**a**^
Illiterate	0	0.0%	1	2.4%	
Elementary	23	46.9%	27	65.9%	
High School	13	26.5%	7	17.1%	
Higher education	13	26.5%	6	14.6%	
**Tumor type**	**56**	**100.0%**	**41**	**100.0%**	**0.210**^**a**^
Infiltrating ductal carcinoma	55	98.2%	37	90.2%	
Invasive lobular carcinoma	1	1.8%	3	7.3%	
In situ ductal carcinoma with microinvasion	0	0.0%	1	2.4%	
Outros	0	0.0%	0	0.0%	
**Histological grade**	**56**	**100.0%**	**41**	**100.0%**	**0.983**
G1	17	30.4%	13	31.7%	
G2	27	48.2%	19	46.3%	
G3	12	21.4%	9	22.0%	
**Immunohistochemistry (hormonal receptors)**	**56**	**100.0%**	**41**	**100.0%**	**0.708**^**a**^
ER + PR +	44	78.6%	31	75.6%	
ER + PR −	3	5.4%	3	7.3%	
ER − PR +	2	3.6%	0	0.0%	
ER − PR −	7	12.5%	7	17.1%	
**Immunohistochemistry (HER2)**	**56**	**100.0%**	**41**	**100.0%**	**0.695**^**a**^
HER 2 −	53	94.6%	38	92.7%	
HER 2 +	3	5.4%	3	7.3%	
**Pathological staging: tumor size (pT)**	**56**	**100.0%**	**41**	**100.0%**	**0.334**^**a**^
T0	0	0.0%	0	0.0%	
Tis*	1	1.8%	0	0.0%	
T1	36	64.3%	22	53.7%	
T2	19	33.9%	19	46.3%	
T3	0	0.0%	0	0.0%	
**Pathological staging: nodes (pN)**	**56**	**100.0%**	**41**	**100.0%**	** < 0.001**^**a**^
N0	0	0.0%	0	0.0%	
N0 i +	1	1.8%	0	0.0%	
N1mi	20	35.7%	1	2.4%	
N1	35	62.5%	30	73.2%	
N2	0	0.0%	8	19.5%	
N3	0	0.0%	2	4.9%	
**Angiolymphatic invasion**	**56**	**100.0%**	**41**	**100.0%**	**0.641**
Yes	26	46.4%	21	51.2%	
No	30	53.6%	20	48.8%	
**Capsular extension**	**56**	**100.0%**	**41**	**100.0%**	** < 0.001**
Yes	5	8.9%	17	41.5%	
No	51	91.1%	24	58.5%	
**Axillary metastasis**	**56**	**100.0%**	**41**	**100.0%**	** < 0.001**^**a**^
Micrometastasis	20	35.7%	1	2.4%	
Macrometastasis	35	62.5%	40	97.6%	
Isolated tumoral cell	1	1.8%	0	0.0%	
**Adjuvant chemotherapy**	**56**	**100.0%**	**41**	**100.0%**	**0.024**
No	14	25.0%	3	7.3%	
Yes	42	75.0%	38	92.7%	
**Radiotherapy**	**56**	**100.0%**	**41**	**100.0%**	**0.080**^**a**^
No	1	1.8%	5	12.2%	
Yes	55	98.2%	36	87.8%	
Tangential fields	39	70.0%	27	66.0%	0.12
Tangential fields and drains	7	12.5%	2	4.9%	
Unknown	9	16.0%	7	17.0%	
**Hormonal therapy**	**56**	**100.0%**	**41**	**100.0%**	**0.896**
No	9	16.1%	7	17.1%	
Yes	47	83.9%	34	82.9%	

Bold values indicates category headings and the total value of the category by group.

*T0* no evidence of primary tumor, *Tis* ductal carcinoma in situ, *T1* tumor size is 2 cm or less across, *T2* tumor size 20–50 mm, *T3* tumor size is more than 5 cm across, *N0 i +*  the area of cancer spread contains fewer than 200 isolated tumor cells and is smaller than 0.2 mm (cancer cells seen in routine stains or immunohistochemistry), *N1mi* micrometastasis to lymph node, *N1* 1–3 lymph nodes affected, *N2 *4–9 lymph nodes affected, *N3* 10 or more lymph nodes affected, *ER* estrogen receptor, *HER2* human epidermal growth factor receptor 2, *PR* progesterone receptor.

*This patient had her whole 0.5 cm invasive tumor removed during percutaneous biopsy. However, she was later classified clinically as T1a.

^a^Chi-squared or Fisher's exact test.

Radiation oncologists at our institution used nomograms to predict the likelihood of metastasis in non-SLNs, and this aided in clinical decision-making. The most commonly used nomograms were from the Memorial Sloan-Kettering Cancer Center^25^ and MD Anderson Cancer Center^26^. Patients who had greater than 30% risk of additional lymph node involvement were treated with drainage radiotherapy^27^ although it is not recommended in the ACOSOG Z0011 protocol. We identified 12.5% and 4.9% (p = 0.12) cases that received drainage radiotherapy in the SLND alone and ALND groups, respectively.

There was a significant reduction in the intraoperative assessment of SLNs after the publication of the ACOSOG Z0011 clinical trial and its adoption as a guideline at our hospital. Of the 415 patients evaluated, 90.2% (46 of 51 patients operated) were subjected to the exam before publication and 30.8% (112 of 364) after. The rate of patients undergoing a second surgery as a result of the anatomopathological result was 3.8%, and the main indications for the second surgical procedure were the presence of three or more positive lymph nodes and gross extranodal disease.

### Overall survival

**Figure 3:**
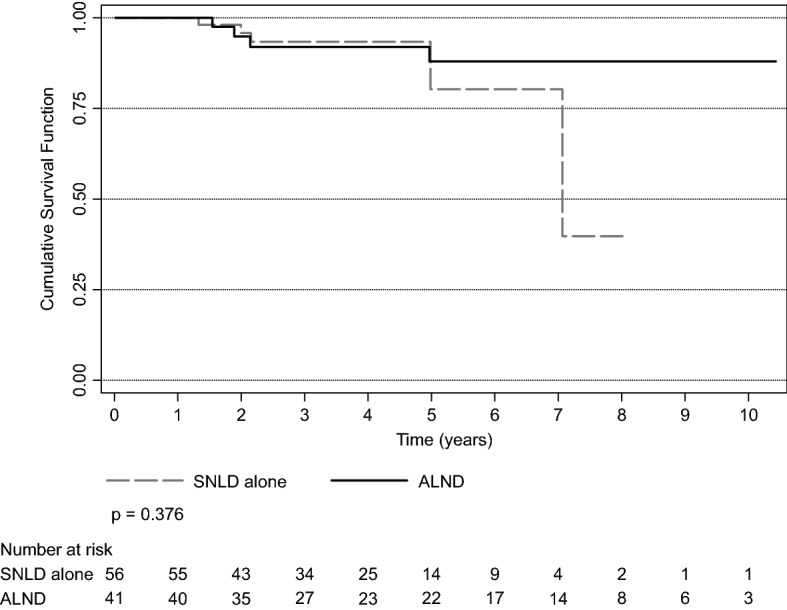
Overall survival over time in patients with breast cancer undergoing ALND or SLND alone. *SLND* sentinel lymph node dissection, *ALND* complete axillary lymph node dissection.

The mean overall survival was 9.18 years (95% confidence interval [CI], 8.47–9.90). There was no difference in survival between the two groups; the 5-year overall survival was 80.1% and 87.5% in the SLND and ALND groups (p = 0.376), respectively (Fig. 3). Only hormonal therapy had a significant effect on survival; patients with positive hormone receptors status lived longer (see Supplementary Table S1). Patients with negative receptors for estrogen (ER) and positive receptor for progesterone (PR) had shorter survival, but only two patients were classified as such; therefore, they were not considered (Table 1). All patients positive for HER2 received targeted therapy, and there was no difference in survival between patients with positive and negative HER2 status. In the survival model, only hormonal therapy had a significant effect (p = 0.018, Table 3), and patients receiving this therapy had a 78% lower risk of death. As in the previous analysis, the two patients who were ER- and PR + were not considered.

**Table 3:** Survival analysis using Cox univariate regression in patients undergoing ALND or SLND alone.

Variable	Gross HR (CI 95%)	p
SLND (reference: ALND)	1.80 (0.48–6.77)	0.386
Age (years)	0.97 (0.92–1.03)	0.307
Tumor size (cm)	1.43 (0.78–2.62)	0.245
**Diagnosis (reference: infiltrating ductal carcinoma)**		0.201
Invasive lobular carcinoma	7.12 (0.83–61.07)	0.073
In situ ductal carcinoma with microinvasion	0.00 (–)	0.987
**Histological grade (reference: G2)**		0.591
G1	0.67 (0.13–3.47)	0.632
G3	1.68 (0.39–7.14)	0.485
Positive lymph nodes	0.99 (0.79–1.24)	0.939
Resected lymph nodes	0.97 (0.88–1.07)	0.512
**Axillary metastasis (reference: macrometastasis)**		0.753
Micrometastasis	1.68 (0.43–6.52)	0.452
Isolated tumoral cell	0.00 (–)	0.991
Capsular extension (reference: no)	2.47 (0.61–10.02)	0.206
Angiolymphatic invasion (reference: no)	1.64 (0.46–5.81)	0.444
**Immunohistochemistry—hormone receptors (reference: ER + PR +)**		0.062
ER + PR −	0.00 (–)	0.989
ER− PR +	16.75 (1.86–150.91)	0.012
ER − PR −	2.87 (0.71–11.51)	0.137
Immunohistochemistry—HER2 (reference: HER 2 -)	0.05 (–)	0.601
Immunohistochemistry (ki67)	0.99 (0.95–1.04)	0.773
Angiolymphatic invasion (reference: no)	1.64 (0.46–5.81)	0.444
Adjuvant chemotherapy (reference: no)	0.32 (0.09–1.13)	0.077
Radiotherapy (reference: no)	0.29 (0.06–1.39)	0.122
Hormonal therapy (reference: no)	0.22 (0.06–0.77)	0.018

(−) not shown due to lack of precision.

*HR *hazard ratio, *Tis* ductal carcinoma in situ, *T1* tumor size, *T2* tumor size 20–50 mm, *N0i +*  the area of cancer spread contains fewer than 200 isolated tumor cells and is smaller than 0.2 mm (cancer cells seen in routine stains or immunohistochemistry), *N1mi* micrometastasis to lymph node, *N1* 1–3 lymph nodes affected, *N2*  4–9 lymph nodes affected, *N3*  10 or more lymph nodes affected, *ER*  estrogen receptor, *PR* progesterone receptor.

The multivariate Cox regression model included the following as predictor variables in the initial model: the intervention group (regardless of their significance), hormonal therapy (significant at 5% in the Cox univariate model), tumour size, the number of positive lymph nodes, the number of resected lymph nodes, capsular extension, the size of axillary metastasis, and adjuvant chemotherapy (different characteristics by intervention group) (Table 4). In this analysis, there was no difference in survival between the groups (p = 0.536) (Table 4). However, the risk of death was 91% lower in patients undergoing hormonal therapy in the final model (p = 0.005) and 86% lower in those treated with adjuvant chemotherapy (p = 0.027). Schoenfeld residuals testing showed that the hazards were proportional in the initial model (p = 0.453) and at the end (p = 0.194), indicating the absence of violation of this assumption.

**Table 4:** Final survival analysis using Cox multivariate regression in patients with breast cancer.

Variables	Initial model		Final model	
	Adjusted HR (95%CI)	p	Adjusted HR (95%CI)	p
SLND (reference: ALND)	1.24 (0.05–29.49)	0.895	1.55 (0.39–6.22)	0.536
Hormonal therapy (reference: no)	0.05 (0.01–0.32)	0.001	0.09 (0.02–0.48)	0.005
Tumor size (cm)	1.18 (0.57–2.46)	0.656	–	–
Positive lymph nodes	0.95 (0.72–1.24)	0.685	–	–
Resected lymph nodes	0.99 (0.79–1.24)	0.918	–	–
Capsular extension (reference: no)	6.52 (0.97–43.73)	0.053	–	–
**Axillary metastasis (reference: macrometastasis)**		0.575		–
Micrometastasis	2.75 (0.42–18.05)	0.293	–	–
Isolated tumoral cell	0.00 (–)	0.99	–	–
Adjuvant chemotherapy (reference: no)	0.17 (0.03–1.13)	0,066	0.14 (0.02–0.8)	0.027

*CI* confidence interval, *HR* hazard ratio, *SLND* sentinel lymph node dissection, *ALND* complete axillary lymph node dissection.

### Locoregional recurrence

**Figure 4:**
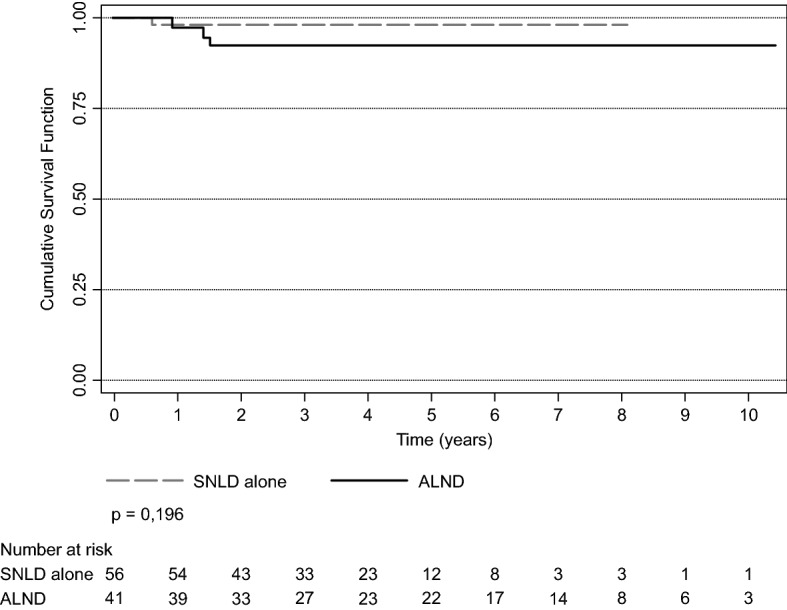
Survival after locoregional recurrence. *SLND* sentinel lymph node dissection, *ALND* complete axillary lymph node dissection.

Locoregional recurrence was a rare event, with only four patients having disease recurrence: 7.3% of patients in the ALND group and 1.7% in the SLND group (p = 0.3075). Recurrence occurred in all cases within 18 months of follow-up. Survival after locoregional recurrence was 10.1 years (95%CI 9.62–10.40), and there was no statistically significant difference between the groups (p = 0.196) (see Supplementary Table S2). Figure 4 shows survival after locoregional recurrence per group.

## Discussion

This study showed that completion of ALND did not improve overall survival or locoregional recurrence in patients with invasive breast cancer and sentinel node metastasis. The similar overall survival between the two groups provides evidence that ALND is unnecessary in patients with up to two metastatic SLNs treated with conservative surgery and radiotherapy. This finding suggests that even in countries such as Brazil, where the overall survival in patients with breast cancer is lower than that in patients in developed nations^23^, conservative surgical treatment of the axilla, in patients who meet Z0011 criteria, is possible. This result corroborates the data from the ACOSOG Z0011 trial^15^.

Patients undergoing SLND alone had a similar survival rate. However, in our study, patients in the SLND group received adjuvant chemotherapy less frequently (75% vs. 92.7% in SLND alone and ALND, respectively, p = 0.024, Table 1). Additionally, they were spared adjuvant chemotherapy because of comorbidities, and these data were not evaluated in the study.

The tendency towards reducing the use of intraoperative lymph node evaluation at our hospital following the publication of the ACOSOG Z0011 trial was similar to that in other studies^6,7,28,29^. Intraoperative assessment of the SLN can be associated with a shorter average time of surgery^6^, a reduction in perioperative costs^30,31^, and a significant increase in the proportion of patients in whom complete dissection can be avoided^32^. According to van der Noordaa et al., intraoperative assessments of the SLN should be performed only in patients with a restricted indication for lymph node dissection in the presence of metastasis in SLN biopsy^33^. Thus, the intraoperative assessment of the SLN is not necessary for patients who meet the ACOSOG Z0011 criteria, and the surgical re-approach resulting from the definitive anatomopathological result of the axilla is rare (3.8%). This is an important finding in our study that can promote the practice of avoiding the intraoperative assessment of SLNs.

Locoregional recurrence was rare, and the rate was similar between the groups. Furthermore, we believe that our follow-up duration was adequate and was suitable for measuring recurrence. Long-term follow-up data from the NSABP trial^19^ showed that recurrence usually occurred early, at 14.8 months on average. In the ACOSOG Z0011 trial^18^, recurrences occurred in 3.1 years, a bit shorter than our average follow-up time of 3.7 years.

The SLND group included postmenopausal women with small tumours (pT1), positive hormone receptors status, and small axillary involvement (35.7% with micrometastasis in the SLN biopsy). These characteristics were similar to those of the same arm in the Z0011 trial^15,18^. However, in the Z0011 study^15^, the arm undergoing complete axillary dissection also had a high prevalence of micrometastases (37.5%), in contrast to that noted in our study.

The ACOSOG Z0011 trial criteria can be considered for patients with HER2 overexpression, triple-negative tumours, and those aged below 50 years. Chung et al. reported no benefit in performing ALND in this subgroup^34^. In our study, the groups were homogeneous in terms of these three variables. The underrepresentation of this group in the ACOSOG Z0011 trial may be due to the local demographic characteristics of patients with breast carcinoma. Nevertheless, it was assumed that the distribution of HER2-positive tumours was balanced between the two arms of the trial.

Several studies around the world have identified increasing acceptance of the Z0011 results and a change in clinical practice in relation to the standard treatment of axillary lymph nodes in patients with breast cancer^35–38^.

A meta-analysis comparing SLND/radiotherapy only with ALND in early-stage breast cancer with limited sentinel node metastasis estimated that overall survival and disease-free survival were higher in the SLND group than in the ALND group, and a greater axillary recurrence was observed in the SLND/radiotherapy group. In conclusion, the omission of ALND in patients with one or two positive SLNs is indicated^39^.

Another meta-analysis of real-world cases evaluating the effects of SLND alone in patients with early-stage breast cancer and one or two positive SLN metastases in the post-Z011 era showed equivalent survival and recurrence outcomes between those undergoing SNLD alone and those undergoing ALND, which demonstrates that SLND alone was safe^40^. However, this shift in clinical practice should not occur in patients with residual lymph node disease following neoadjuvant chemotherapy^41^. All these studies included patients who were treated with systemic adjuvant therapy.

Complete ALND might be an overtreatment for many patients with capsular extravasation in the dissected SLNs. The Z0011 trial excluded patients with gross capsular extravasation and did not analyse the effect of microscopic capsular extravasation on recurrence or survival, making the management of these patients uncertain^15,18^. The extension of capsular extravasation is directly associated with the burden of axillary disease^42^. However, the rates of local, regional, or distant recurrence and mortality were similar between patients with and without capsular extravasation of ≤ 2 mm^43^, and regional recurrence was rare and was similar to that in patients without capsular extravasation even in the absence of nodal radiotherapy. Capsular extravasation is not the only reason for complete ALND^44^. In our study, we identified five patients with capsular extravasation of ≤ 2 mm who were treated with SLND alone, avoiding the morbidity associated with complete axillary resection. However, these patients received regional radiotherapy at our hospital.

We acknowledge that translating the Z0011 results into clinical practice is complicated due to inconsistent use of radiotherapy fields in their study. In a prospective study of 793 patients with SLN metastasis, using the ACOSOG Z0011 eligibility criteria resulted in the avoidance of ALND in 84% of patients, and the 5-year cumulative regional recurrence rate was 1%, which did not differ between radiotherapy fields. The authors concluded that even without the routine use of nodal radiotherapy, complete dissection could be avoided with excellent regional control^45^. Hopefully, we will have answers about the real influence of radiotherapy in regional control with the results of the ongoing trials^46–49^.

This was a retrospective study based on the medical records; thus, we were unable to evaluate costs and surgical times after the change in the clinical approach in our hospital after the publication of the ACOSOG Z0011 study. Studies that evaluated cost reduction associated with the elimination of complete axillary dissection^30,31^, did not consider the risk of surgical re-approach due to the presence of more than two SLNs with macrometastasis or capsular extravasation. The cost of a second surgery remains to be evaluated. Even the ACOSOG Z0011 trial did not report the rate of surgical re-approach in the group subjected to SLND alone. The rate of surgical re-approach in this study was very low and answered this question. In addition, this was the first study in our country to address the findings of Z0011 implementation, which was important to encourage conservative surgical treatment of the axilla in our country and other developing countries, with the aim of disseminating this practice and benefiting patients.

The preliminary internal evaluation of the results of this study prompted major changes in our hospital’s clinical approach, with more conservative surgeries being performed and the elimination of ultrasonography, the findings of which would often cause patients to undergo radical lymphadenectomy in the absence of SLN biopsy results in the past.

## Conclusions

The overall survival and locoregional recurrence in patients with metastatic axillary SLNs treated with SLND were similar to those in patients treated with complete ALND. The elimination of routine axillary lymphonodectomy and the implementation of SLND in women who met the ACOSOG Z0011 criteria at our hospital benefited the patients who could be treated with less aggressive surgery. The de-escalation of ALND to SLND in women with up to two metastases in the SLN treated with conservative surgery and radiotherapy is possible. Our study showed that the ACOSOG Z0011 recommendation is feasible even in developing countries.

## Supplementary Information

Supplementary Tables.

## Data Availability

The datasets used and/or analyzed during the current study are available from the corresponding author upon reasonable request.
