# Supplementary material for: Survival and recurrence with or without axillary dissection in patients with invasive breast cancer and sentinel node metastasis
Source: Sci Rep. 2021 Oct 6;11:19893. doi: 10.1038/s41598-021-99359-w (PMC8494764; doi:10.1038/s41598-021-99359-w)
Supplement: Supplementary file 1 — Supplementary Tables. [file 41598_2021_99359_MOESM1_ESM.docx]

**Supplementary Information**

**“Survival and recurrence with or without axillary dissection in patients with invasive breast cancer and sentinel node metastasis”**

**Supplementary Table S1.** Kaplan-Meier survival analysis in patients undergoing ALND or SLND alone

| **Death** | **Accumulated % of survival** | | | | **p*** |  |  |  |  |  |  |
| --- | --- | --- | --- | --- | --- | --- | --- | --- | --- | --- | --- |
|  | **1 year** | **2 years** | **5 years** | **10 years** |  |  |  |  |  |  |  |
| **Total** | 100.0 | 95.2 ± 2.3 | 84.9 ± 5.0 | 79.3 ± 7.2 | - |  |  |  |  |  |  |
| **Group** |  |  |  |  | 0.376 |  |  |  |  |  |  |
| SLND alone | 100.0 | 95.8 ± 2.9 | 80.1 ± 9.3 | - |  |  |  |  |  |  |  |
| ALND | 100.0 | 94.5 ± 3.8 | 87.5 ± 6.0 | 87.5 ± 6.0 |  |  |  |  |  |  |  |
| **Age** |  |  |  |  | 0.722 |  |  |  |  |  |  |
| ≤ 50 years old | 100.0 | 89.5 ± 5.7 | 89.5 ± 5.7 | - |  |  |  |  |  |  |  |
| 51 years or older | 100.0 | 98.0 ± 1.9 | 81.8 ± 7.2 | 71.6 ± 11.5 |  |  |  |  |  |  |  |
| **Race** |  |  |  |  | 0.722 |  |  |  |  |  |  |
| White | 100.0 | 96.8 ± 2.3 | 86.2 ± 5.7 | 78.4 ± 9.1 |  |  |  |  |  |  |  |
| Not white | 100.0 | 90.2 ± 6.6 | 81.2 ± 10.4 | - |  |  |  |  |  |  |  |
| **Educational level** |  |  |  |  |  |  |  |  |  |  |  |
| Illiterate | 100.0 | 100.0 | - | - | 0.938 |  |  |  |  |  |  |
| Elementary | 100.0 | 95.7 ± 3.0 | 83.1 ± 6.5 | 83.1 ± 6.5 |  |  |  |  |  |  |  |
| High School | 100.0 | 94.4 ± 5.4 | 88.1 ± 7.9 | 88.1 ± 7.9 |  |  |  |  |  |  |  |
| Higher education | 100.0 | 91.7 ± 8.0 | 91.7 ± 8.0 | - |  |  |  |  |  |  |  |
| **Tumor type** |  |  |  |  | 0.096 |  |  |  |  |  |  |
| Infiltrating ductal | 100.0 | 95.0 ± 2.4 | 85.7 ± 5.1 | 79.5 ± 7.6 |  |  |  |  |  |  |  |
| Invasive lobular carcinoma | 100.0 | 100.0 | - | - |  |  |  |  |  |  |  |
| In situ ductal carcinoma with microinvasion | 100.0 | 100.0 | 100.0 | - |  |  |  |  |  |  |  |
| **Histological grade** |  |  |  |  | 0.571 |  |  |  |  |  |  |
| G1 | 100.0 | 96.2 ± 3.8 | 92.3 ± 5.2 | 92.3 ± 5.2 |  |  |  |  |  |  |  |
| G2 | 100.0 | 97.5 ± 2.5 | 85.6 ± 6.9 | - |  |  |  |  |  |  |  |
| G3 | 100.0 | 89.4 ± 7.1 | 74.5 ± 14.8 | - |  |  |  |  |  |  |  |
| **Immunohistochemistry (hormone receptors)** | |  |  |  | 0.006 |  |  |  |  |  |  |
| ER + PR + | 100.0 | 98.4 ± 1.6 | 88.7 ± 5.1 | 81.3 ± 8.5 |  |  |  |  |  |  |  |
| ER + PR - | 100.0 | 100.0 | 100.0 | 100.0 |  |  |  |  |  |  |  |
| ER - PR + | 100.0 | 50.0 ± 35.4 | - | - |  |  |  |  |  |  |  |
| ER - PR - | 100.0 | 82.5 ± 11.3 | 68.8 ± 15.7 | - |  |  |  |  |  |  |  |
| **Immunohistochemistry (HER2)** |  |  |  |  | 0.432 |  |  |  |  |  |  |
| HER 2 - | 100.0 | 94.9 ± 2.5 | 84.0 ± 5.3 | 78.4 ± 7.3 |  |  |  |  |  |  |  |
| HER 2 + | 100.0 | 100.0 | 100.0 | - |  |  |  |  |  |  |  |
| **Pathological staging: tumor size (pT)** |  |  |  |  | 0.912 |  |  |  |  |  |  |
| Tis | 100.0 | 100.0 | - | - |  |  |  |  |  |  |  |
| T1 | 100.0 | 92.1 ± 3.8 | 88.6 ± 5.0 | 79.7 ± 9.5 |  |  |  |  |  |  |  |
| T2 | 100.0 | 100.0 | 74.5 ± 12.4 | - |  |  |  |  |  |  |  |
| **Pathological staging: nodes (pN)** |  |  |  |  | 0.705 |  |  |  |  |  |  |
| N0 i+ | 100.0 | 100.0 | 100.0 | - |  |  |  |  |  |  |  |
| N1mi | 100.0 | 94.1 ± 5.7 | 88.2 ± 7.8 | - |  |  |  |  |  |  |  |
| N1 | 100.0 | 96.4 ± 2.5 | 86.6 ± 6.1 | 86.6 ± 6.1 |  |  |  |  |  |  |  |
| N2 | 100.0 | 87.5 ± 11.7 | 70.0 ± 18.2 | - |  |  |  |  |  |  |  |
| N3 | 100.0 | 100.0 | - | - |  |  |  |  |  |  |  |
| **Angiolymphatic invasion** |  |  |  |  | 0.435 |  |  |  |  |  |  |
| No | 100.0 | 93.0 ± 3.9 | 88.1 ± 6.0 | 88.1 ± 6.0 |  |  |  |  |  |  |  |
| Yes | 100.0 | 97.4 ± 2.5 | 81.3 ± 8.2 | - |  |  |  |  |  |  |  |
| **Capsular extension** |  |  |  |  | 0.188 |  |  |  |  |  |  |
| No | 100.0 | 95.5 ± 2.6 | 88.0 ± 4.9 | 82.1 ± 7.3 |  |  |  |  |  |  |  |
| Yes | 100.0 | 94.1 ± 5.7 | 65.9 ± 20.0 | - |  |  |  |  |  |  |  |
| **Axillary metastasis** |  |  |  |  | 0.682 |  |  |  |  |  |  |
| Micrometastasis | 100.0 | 94.1 ± 5.7 | 88.2 ± 7.8 | - |  |  |  |  |  |  |  |
| Macrometastasis | 100.0 | 95.4 ± 2.6 | 84.1 ± 6.0 | 84.1 ± 6.0 |  |  |  |  |  |  |  |
| Isolated tumoral cell | 100.0 | 100.0 | 100.0 | - |  |  |  |  |  |  |  |
| **Adjuvant chemotherapy** |  |  |  |  | 0.059 |  |  |  |  |  |  |
| No | 100.0 | 92.9 ± 6.9 | 57.1 ± 17.6 | 57.1 ± 17.6 |  |  |  |  |  |  |  |
| Yes | 100.0 | 95.7 ± 2.4 | 91.1 ± 4.1 | 83.5 ± 8.2 |  |  |  |  |  |  |  |
| **Radiotherapy** |  |  |  |  | 0.097 |  |  |  |  |  |  |
| No | 100.0 | 100.0 | 66.7 ± 19.2 | - |  |  |  |  |  |  |  |
| Yes | 100.0 | 94.8 ± 2.5 | 86.5 ± 5.2 | 80.3 ± 7.6 |  |  |  |  |  |  |  |
| **Hormonal therapy** |  |  |  |  | 0.009 |  |  |  |  |  |  |
| No | 100.0 | 75.0 ± 12.7 | 60.0 ± 16.8 | - |  |  |  |  |  |  |  |
| Yes | 100.0 | 98.5 ± 1.5 | 89.4 ± 4.8 | 82.5 ± 8.0 |  |  |  |  |  |  |  |
| Tis = ductal carcinoma in situ; T2 = tumor size 20-50 mm. N0i+ = the area of cancer spread contains fewer than 200  isolated tumor cells and is smaller than 0.2 mm (cancer cells seen in routine stains or immunohistochemistry);  N1mi = micrometastasis to lymph node; N1 = 1-3 lymph nodes affected; N2 = 4-9 lymph nodes affected;  N3 = 10 or more lymph nodes affected; ER = estrogen receptor; PR = progesterone receptor.  HER2 = human epidermal growth factor receptor 2; SLND = sentinel lymph node dissection;  ALND = complete axillary lymph node dissection. | | | | | | |  |  |  |  |  |

**Supplementary Table S2.** Kaplan-Meier survival analysis of locoregional disease recurrence in patients undergoing ALND or SLND alone

|  | **Accumulated % of survival** | | | | | | | **p*** | |  |  |
| --- | --- | --- | --- | --- | --- | --- | --- | --- | --- | --- | --- |
|  | **1 year** | **2 years** | | **5 years** | | **10 years** | |  |  |  |  |
| **Total** | 97.9 ± 1.5 | 95.6 ± 2.1 | | 95.6 ± 2.1 | | 95.6 ± 2.1 | | - | |  |  |
| **Group** |  |  | |  | |  | | 0.196 | |  |  |
| SLND alone | 98.2 ± 1.8 | 98.2 ± 1.8 | | 98.2 ± 1.8 | | - | |  | |  |  |
| ALND | 97.5 ± 2.5 | 92.3 ± 4.3 | | 92.3 ± 4.3 | | 92.3 ± 4.3 | |  | |  |  |
| *Log rank test |  | |  | |  | |  | |  | |  |
| SLND = sentinel lymph node dissection; ALND = complete axillary lymph node dissection | | | | | | | | | | | |
